# Evaluation of clopidogrel impact on canine platelet function using flow cytometry and thromboelastography platelet mapping

**DOI:** 10.3389/fvets.2025.1555641

**Published:** 2025-06-05

**Authors:** Eunchae Yoon, Chaewon Shin, Hyeona Bae, Kyu-Woan Cho, Dong-In Jung, Jinho Park, Dongbin Lee, DoHyeon Yu

**Affiliations:** ^1^College of Veterinary Medicine, Gyeongsang National University, Jinju, Republic of Korea; ^2^College of Veterinary Medicine, Jeonbuk National University, Iksan, Republic of Korea

**Keywords:** clopidogrel, flow cytometry, platelet aggregation, thromboelastography, adenosine diphosphate

## Abstract

**Background:**

Clopidogrel is frequently used in veterinary medicine to therapeutically decrease platelet function, although some different dosages have been published. Therefore, we assessed the antiplatelet effects of the recommended dosage (1 mg/kg PO q24h) on canine platelet function.

**Methods:**

Five dogs were administered either clopidogrel or placebo, with a 14-day washout period. Platelet function was assessed using thromboelastography (TEG) and flow cytometry, complete blood count, and biochemical analyses were performed for clinicopathological evaluation. Blood samples were collected at baseline and 7 days after drug administration. TEG parameters including maximum amplitude and platelet mapping for adenosine diphosphate (ADP)-induced responses were used to monitor therapeutic efficacy. Flow cytometry was used to analyze CD62P expression and platelet activation stimulated by ADP and other agonists.

**Results:**

TEG analysis demonstrated a significant reduction in ADP-induced clot strength following clopidogrel administration (*p* < 0.05), indicating effective platelet inhibition. Flow cytometry confirmed the marked inhibition of platelet activation, with significant decreases in the percentage of CD62P positive platelets and the mean fluorescence intensity under ADP and epinephrine stimulation (*p* < 0.05). Hematological and biochemical parameters remained stable across all groups, confirming the safety of clopidogrel administration. These findings highlight the efficacy and safety of clopidogrel as an antiplatelet agent in dogs.

**Conclusion:**

This study confirmed the efficacy of low-dose (1 mg/kg, p.o., q24h) clopidogrel in dogs without a loading dose. TEG and flow cytometry are effective tools for assessing clopidogrel responsiveness in dogs and may aid in optimizing antiplatelet therapy in clinical practice.

## 1 Introduction

For normal hemostasis, the rapid recruitment of platelets to regions with vascular injury, by forming a quick platelet plug, is crucial for preventing bleeding. Platelet defects can lead to bleeding. However, decreasing platelet reactivity may be indicated in animals at increased risk of thrombotic disease, such as those with corticosteroid administration, dirofilariasis, disseminated intravascular coagulation, hyperadrenocorticism, neoplasia, protein-losing enteropathy, and sepsis (1). Thus, effective antiplatelet treatment is essential for prevention.

Clopidogrel, an orally administered prodrug of the thienopyridine class, causes irreversible inhibition of the adenosine diphosphate (ADP) receptor P2Y12 on platelets (2). The prodrug is converted in the liver to a thiol-containing compound that irreversibly binds to cysteine residues on P2Y12, inhibiting platelet aggregation. Clopidogrel resistance, referring to an insufficient antiplatelet response despite appropriate dosing as assessed by platelet function tests, has been reported in approximately 30% of human patients (3, 4). Various mechanisms have been proposed to explain this lack of response, including genetic polymorphisms in the P2Y12 receptor and metabolism by liver enzymes, specifically cytochrome P450 enzymes. This variability has led to discussions about personalized monitoring to gauge the level of platelet inhibition and improve patient outcomes (5). In veterinary medicine, the recommended dosage of clopidogrel is 1 mg/kg, but there may be variability in drug effects (2). In this study, platelet function in dogs after treatment with clopidogrel was assessed using flow cytometry and TEG, rather than previous studies, which used platelet aggregometry or the PFA-200 platelet function analyzer (2). No medications are currently approved for use in companion animals, although oral antiplatelet drug protocols have been extensively researched in humans, and few studies are available to establish reliable guidelines (6). Platelet inhibition following treatment with clopidogrel or aspirin may vary (as observed in humans) and warrant platelet function testing to monitor treatment response (2). In veterinary medicine, a study assessing platelet function found that only one-third of dogs experienced complete inhibition of platelet aggregation when administered aspirin at a dose of 1 mg/kg/day for 10 days (7).

Platelets have several functions that require reliable detection, and various tests have been optimized for each function. In this study, platelet function was assessed using an alternative function test and a viscoelastic test rather than the employed transmission aggregometry, which is commonly used to guide antiplatelet therapy.

Thromboelastography (TEG) is a patient-side viscoelastic test that provides a comprehensive assessment of hemostatic potential and a graphical depiction of clot formation over time (8). Platelet mapping can be incorporated into TEG to enhance the evaluation of platelet function, which plays a critical role in thrombotic disorders (9). In human medicine, platelet mapping TEG has been widely used to assess responses to antiplatelet therapy (10). However, studies using TEG for monitoring antiplatelet response in veterinary medicine are limited, with few studies evaluating the effect of clopidogrel on platelet mapping TEG in healthy dogs (2, 8, 11). Moreover, it is unknown whether platelet mapping TEG can allow for the evaluation of the impact of specific antiplatelet agents on coagulation (12).

Flow cytometry offers a unique opportunity for multiparametric single-cell analysis and is a valuable tool for evaluating platelet function (13). It enables studies of various aspects of platelet function in response to different platelet agonists. This can be performed using only a small volume of whole blood and blood with low platelet counts (PLTs) (14). In human studies, it is increasingly being used to evaluate the activation state of circulating platelets in patients and monitor antiplatelet therapy (15).

We hypothesized that clopidogrel would achieve sufficient antiplatelet effects even without a loading dose. This was measured using a range of platelet function assessments, such as platelet mapping TEG and flow cytometry. Additionally, in this study, we aimed to perform platelet-mapping TEG and flow cytometry in healthy dogs following clopidogrel administration to evaluate their utility for monitoring treatment responses.

## 2 Materials and methods

### 2.1 Experimental animals

Five neutered male Beagles were used in this study. None of the dogs received any medication or treatment for 2 weeks before the initiation of the study. Their normal health statuses were confirmed by physical examination, complete blood count (CBC), and biochemical analysis before inclusion in the study. The mean ± standard deviation (SD) of the ages and body weights of the dogs were 4 ± 0.20 years and 9 ± 0.62 kg, respectively.

Throughout the drug administration period, all the dogs were closely monitored for adverse effects. The dogs were fed dry food once a day and had easy access to water. The dogs were fasted for ≥ 12 h before blood sample collection. The animal experiments were approved by the Institutional Animal Care and Use Committee (GNU-190226-D0012).

### 2.2 Study design

Initially, five dogs were randomly assigned to either the clopidogrel (*n* = 3) or placebo (*n* = 2) groups. For the clopidogrel group, clopidogrel was administered orally for 7 days, followed by 14 days without any treatment (16). The dosage was calculated based on the weight of each dog. Following the washout period, the dogs switched treatments: the two dogs originally in the clopidogrel group received a placebo, and the two dogs originally in the placebo group received clopidogrel. One dog underwent two trials with clopidogrel.

### 2.3 Drug administration

Dogs in the clopidogrel group received 1 mg/kg p.o., q24h of 75-mg clopidogrel (Plavix Tablet; Alvogen Korea Co., Ltd., South Korea), which was ground into a powder, and the dose was calculated for each dog. The powder was transferred to empty gelatin capsules. The drug was administered at approximately the same time daily.

### 2.4 Blood sample collection

Blood samples were collected to confirm the anti-platelet effects of clopidogrel. Samples were obtained before treatment (Day 0) as baseline and after 7 consecutive days (Day 7) of drug administration. In the clopidogrel group, samples were collected 3 h after drug administration. For the placebo group, samples were collected simultaneously (2).

Using 21 G butterfly needles (BD Vacutainer® Safety-Lok™, Becton Dickinson, Franklin Lakes, NJ, USA), blood samples were carefully collected via jugular venipuncture with minimum stasis. Approximately 2 mL of blood was discarded to ensure that atraumatic venipuncture was performed. The blood samples were collected into 1 citrated (Greiner Bio-One Vacuette® Sodium Citrate, Greiner Bio-One, Kremsmünster, Austria), 1 EDTA (BD Vacutainer® K2 EDTA, Becton Dickinson, Franklin Lakes, NJ, USA), and 2 heparin vacutainer plastic tubes (BD Vacutainer® Lithium Heparin, Becton Dickinson, Franklin Lakes, NJ, USA) in that order. All tubes were inverted approximately four times immediately after the blood was collected into the tubes. The citrated samples were used for platelet function analysis and TEG; they contained 3.2% sodium citrate to yield a 9:1 blood-to-citrate ratio. EDTA blood samples were used for CBC, fibrinogen concentration test, and PLT. The first heparin blood sample was used for biochemical analysis, and the second for platelet mapping TEG (17, 18).

### 2.5 TEG

All sample analyses were conducted using a single machine (TEG 5000 hemostasis analyzer; Haemoscope Corporation), according to the manufacturer’s instructions. Citrated whole-blood samples were allowed to rest at room temperature for 30 min before analysis to prevent time-dependent differences. The TEG cup was pre-warmed at 37°C approximately 5 min before the analysis. Citrated blood samples were activated with kaolin by aliquoting 1 mL of citrated blood into commercial kaolin vials and gently inverting the vials 5 times. Twenty microliters of 0.2 M calcium chloride was filled in a TEG cup. Kaolin-activated citrated blood (340 μL) was added to make up a total volume of 360 μL in the TEG cup.

The following eight TEG parameters were measured in each assay: split point (SP), reaction time (R), clot kinetics value (K), *α*-angle, maximum amplitude (MA), lysis at 30 min (LY30), G (global clot strength), and coagulation index (CI). The SP value, which assesses the ability of platelets to aggregate without any external activators, refers to spontaneous platelet aggregation. The R value represents the clotting time until initial clot formation. The K value represents the clot formation duration from 2 mm to 20 mm. The *α*-angle value, which is the tangent between the baseline and TEG curve, represents the acceleration of cross-linking and formation of fibrin. The MA value represents the ultimate strength of the clot, which indicates the maximum dynamic properties of platelet and fibrin bonding. LY30 is the TEG amplitude at 30 min after MA, denoting the extent of fibrinolysis (19). The G and CI were calculated by the investigators. G is automatically calculated using the following equation (18):

$$G=5000\times \frac{\mathrm{MA}}{100-\mathrm{MA}}$$

The CI was calculated as follows:

CI=−0.245R+0.0184K+0.1655MA−0.0241α−5.220

### 2.6 Platelet mapping TEG

Heparinized blood and platelet mapping kits were used to evaluate platelet responses to ADP. Both channels in a single machine were operated simultaneously before initiating citrated blood TEG. For channel 1, 10 μL of the activated F reagent was added to a pre-warmed TEG cup, followed by 360 μL of heparinized blood to measure MA_fibrin_. Channel 1 tracing was run to measure the strength of the cross-linked fibrin clot alone. For channel 2, 10 μL of the activated F reagent was added to a pre-warmed TEG cup, followed by ADP reagent to measure MA_ADP_. Subsequently, the TEG cup for channel 2 was filled with 360 μL of heparinized blood for the final concentration of 2 μM ADP. Channel 2 tracing was performed to determine the clot strength generated by ADP-induced platelet activity.

**Figure 1:**
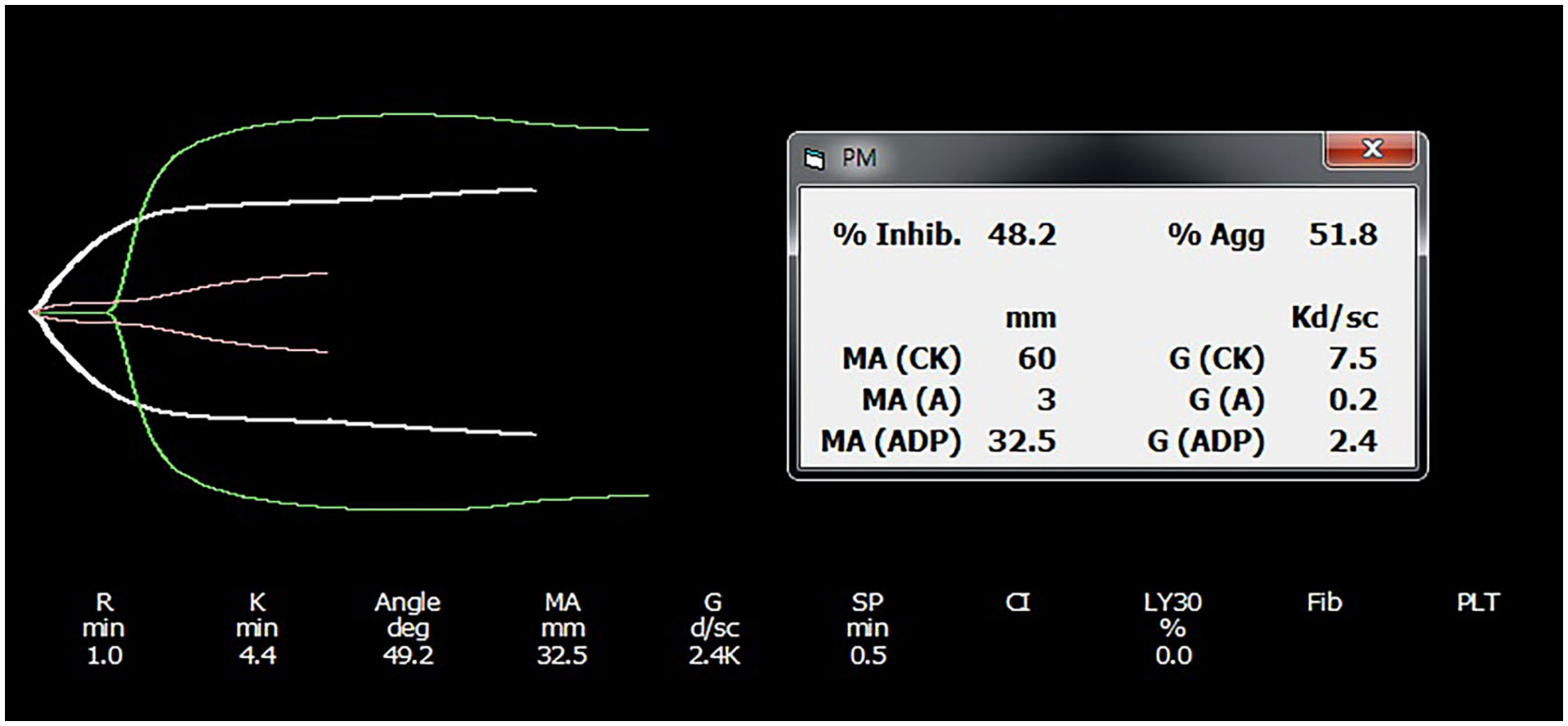
Representative platelet mapping TEG tracing result. TEG platelet mapping tracings from an individual in the clopidogrel group are provided, with results shown before and after drug administration. Each tracing was obtained using citrated TEG analysis. The TEG software calculates the contribution of ADP-induced platelet activation to maximal amplitude using the following formula: ([MA_ADP_ – MA_fibrin_/MA_thrombin_ – MA_fibrin_] × 100) and subtracts this value from 100 to derive percentage platelet inhibition. TEG, thromboelastography; MA, maximum amplitude.

Each trace was compared using citrated TEG analysis (Figure 1). The difference in MA was used for therapeutic monitoring of clopidogrel in dogs. The thrombelastograph software calculated the contribution of ADP-induced platelet activation to MAs as follows:

$$\text{contribution}=\frac{MA_{\mathrm{ADP}}-MA_{\text{fibrin}}}{MA_{\text{thrombin}}-MA_{\text{fibrin}}}\times 100$$

The calculated value was subtracted from 100 to derive the percentage platelet inhibition (8). MA_thrombin_ utilized the MA value from the previous TEG and was conducted with kaolin-activated citrated blood.

### 2.7 Flow cytometry analysis of platelets

All procedures were conducted within 30 min of blood sampling. The optimal antibody and agonist concentrations were determined based on the largest difference in the mean fluorescence intensity between the isotype and samples that had a positive response before analysis. Twenty microliters of diluted citrated blood with a modified HEPES-Tyrode’s buffer (0.14 M NaCl, 2.7 mM KCl, 1 mM MgCl_2_, 12 mM NaHCO_3_, 0.4 mM NaHPO_4_, 5.5 mM glucose, 10 mM HEPES, and 0.5% BSA, pH 7.4), yielding a blood to buffer ratio of 1:5 immediately after blood collection, was mixed with 80 μL of agonist and fluorescein isothiocyanate (FITC) anti-human CD62P antibody (1E3 clone; Santa Cruz biotech, Santa Cruz, California, USA) and allophycocyanin (APC) anti-human CD61 antibody (VI-PL2 clone; Bio-legend, San Diego, USA) cocktail (17). Isotype control (FITC-conjugated mouse IgG; Santa Cruz Biotech) was used for each analysis. For platelet stimulation, 12.5 μg/mL collagen (Chrono Log Corp, Havertown, Pennsylvania), 20 μM ADP (Chrono Log Corp, Havertown, Pennsylvania), and 20 μM epinephrine (Chrono Log Corp, Havertown, Pennsylvania) were used as agonists. Mixtures to a final of 100 μL were incubated for 35 min in a water bath at 37°C. To stop incubation, 800 μL of FACS Lysing solution (BD Bioscience, San Jose, CA, USA) was added to disrupt the red blood cells, prevent interference with light scattering, and fix samples for 15 min at room temperature. All tubes were centrifuged at 400 × g at room temperature for 5 min three times after incubation. For each spin, the supernatant liquid was discarded and platelets were resuspended in phosphate-buffered saline (PBS) (BioWhittaker, Lonza, Belgium). Tubes were stored at 4°C until analysis. The analysis was performed within 3 h of sample preparation. The storage duration did not affect the results until 24 h after fixation.

The platelets were evaluated using a flow cytometer (BD FACSCalibur, BD Biosciences). Data were analyzed using the FlowJo software (Ashland, Oregon, USA). To identify platelets, platelet populations were gated using a combination of light scattering and CD61-APC fluorescence. The threshold was set depending on the CD61 APC; therefore, only CD61 APC-positive events were included in the analysis. A histogram was plotted with a log value for CD62P FITC on the x-axis and PLT on the y-axis. Platelet function was expressed as a percentage of the mean fluorescence intensity (MFI) of CD62P FITC-positive platelets (17) (Supplementary Figure 1).

### 2.8 Clinicopathologic analysis

TEG measurements are influenced by blood components and require clinicopathological analysis. CBCs was performed using automatic analyzers (IDEXX ProCyte Dx® Hematology Analyzer; IDEXX Laboratories, Inc., Westbrook, ME, USA), and they included hematocrit (HCT, %), PLT (10^9^/L), and white blood cell count (WBC, 10^9^/L). To confirm this, manual packed cell volume (PCV) and PLTs were obtained from each sample. On measuring the fibrinogen levels, manual PCV was performed using a plasma microhematocrit capillary tube. A small drop of EDTA-anticoagulated blood was stained using a Diff-Quik staining kit (Siemens Healthineers, Deerfield, IL, USA) and manually examined for platelets. PLT was estimated by averaging the number of platelets in five fields of the monolayer using a phase contrast microscope (ZEISS Axio Scope A1; Carl Zeiss AG, Oberkochen, Germany).

A serum chemistry test was conducted using a heparin tube and analyzed by an automated chemistry analyzer (Catalyst One® Chemistry Analyzer, IDEXX Laboratories, Inc., Westbrook, ME, USA). The measured parameters included alanine aminotransferase (ALT, U/L), alkaline phosphatase (ALP, U/L), and gamma-glutamyl transferase (GGT, U/L) levels, which serve as indicators of hepatic function relevant to clopidogrel metabolism.

Plasma fibrinogen was measured with the heat precipitation method (20). EDTA anti-coagulant blood was drawn into two micro-hematocrit capillary tubes. One side of each tube was filled with clay and the tubes were centrifuged in a microhematocrit for 5 min. One of the tubes was carefully placed in a water bath at 56°C (± 1°C) for 3 min. The entire plasma portion of the tube was maintained under the water surface. The plasma became opaque due to fibrinogen precipitation during this procedure. The tubes were centrifuged for 5 min after incubation. The serum tube contained fibrinogen precipitated above the buffy coat. The length of the precipitate column was measured and compared with the length of the plasma column. The ratio of the plasma length to the fibrinogen length was used to represent the fibrinogen quantity.

### 2.9 Statistical analysis

Statistical analyses were performed using SPSS (version 27.0.0; IBM Co., Armonk, NY, USA) and GraphPad Prism version 8.0.2 (GraphPad Software Inc., San Diego, CA, USA). The data were presented as the mean ± SD, and their normality was evaluated using the Shapiro–Wilk test. Differences between the clopidogrel and placebo groups over time were analyzed using two-way analysis of variance, focusing on main and interaction effects. *Post-hoc* analyses were conducted using Sidak’s multiple comparison test. Statistical significance was set at *p* < 0.05, with *p*-values of < 0.01 interpreted as highly significant.

## 3 Results

### 3.1 Clinical observations after clopidogrel administration in dogs

All the dogs remained clinically healthy throughout the study period and tolerated clopidogrel. There were no signs of petechiae, bruising, or hemorrhage, nor was there any evidence of hematoma formation at the venipuncture sites in any dog.

### 3.2 Kaolin-activated TEG and platelet mapping

**Figure 2:**
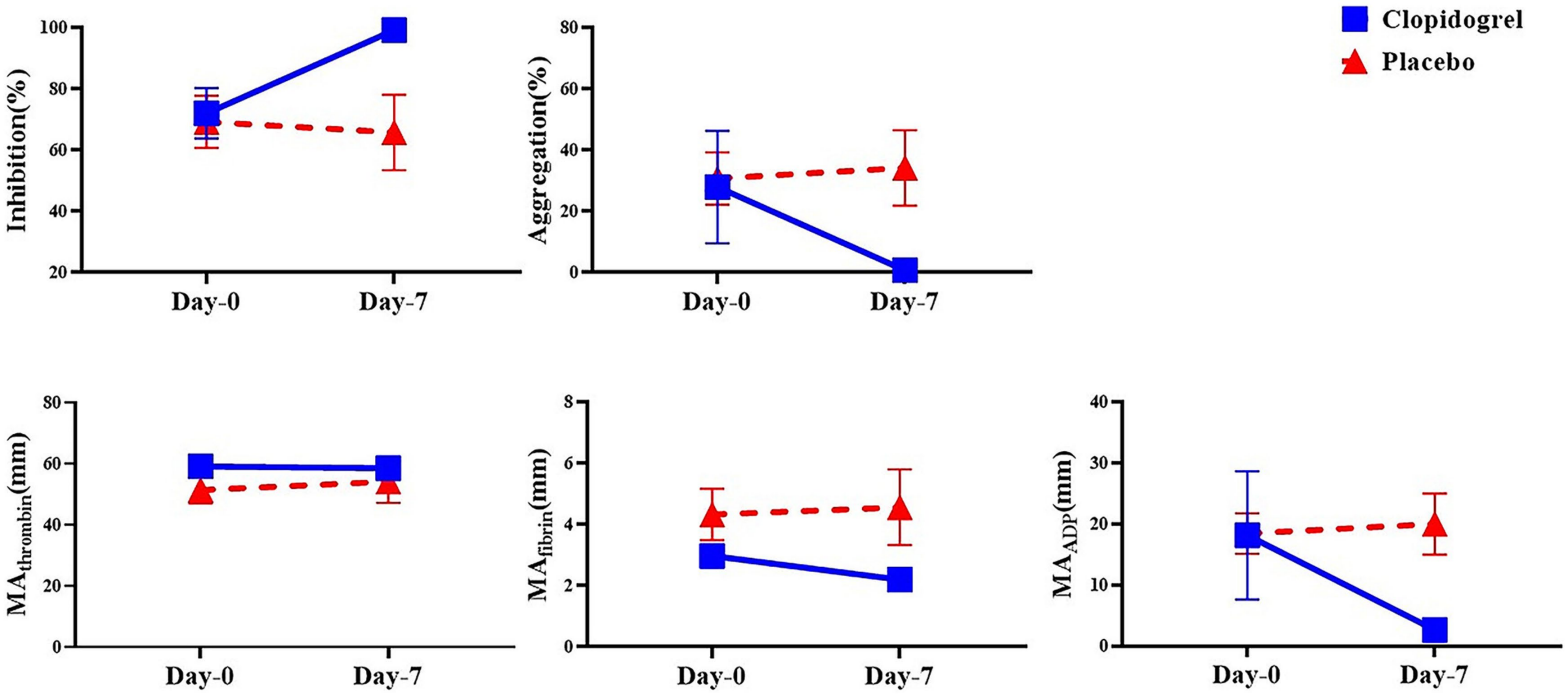
The mean of the platelet mapping TEG parameter within groups over time. This figure shows the mean values of platelet mapping TEG parameters in the clopidogrel (solid line) and placebo (dashed line) groups on Days 0 and 7. Parameters measured include inhibition (%) and aggregation (%) to assess platelet inhibition and aggregation, respectively, along with MA values attributed to thrombin, fibrin, and ADP (MA_thrombin, MA_fibrin, and MA_ADP), which reflect platelet function in response to different activation pathways. * *p* < 0.05, indicating a significant difference between results for days 0 and 7 within each group. TEG, thromboelastography; MA, maximum amplitude; ADP, adenosine diphosphate.

No significant differences were detected between the clopidogrel and placebo groups on days 0 and 7 for the kaolin-activated TEG parameters (SP, R, K, angle, LY30, and CI), as shown in Table 1. All parameters remained within reference ranges, and no significant changes were observed within each group over time. However, platelet mapping revealed a rapid inhibitory effect on ADP-induced clot strength. Comparisons of the day points within each group (Figure 2) revealed significant differences in mean MA_ADP_ for the clopidogrel-treated dogs. The mean ± SD MA_ADP_ for the clopidogrel-treated dogs decreased significantly from 18.16 ± 10.50 mm on day 0 to 2.64 ± 0.53 mm on day 7. In contrast, the corresponding values for the placebo group increased from 18.43 ± 5.76 mm on day 0 to 20.00 ± 8.68 mm on day 7. However, this increase was not statistically significant. Furthermore, the mean ± SD difference in MA_fibrin_ was 2.98 ± 0.68 mm on day 0 and 2.20 ± 0.16 mm on day 7 for the clopidogrel-treated dogs. The placebo group values were 4.33 ± 1.46 mm on day 0 and 4.57 ± 2.14 mm on day 7. Overall, the values in the placebo group were higher than those in the clopidogrel-treated group, but no statistical significances were observed between the groups. Furthermore, there were no significant differences in the values across the days in either group.

**Table 1:** Results of TEG parameters before (Day 0) and after (Day 7) treatment in the clopidogrel and placebo groups.

Variable	Clopidogrel		Placebo		*p*-value
	Day 0	Day 7	Day 0	Day 7	
SP (min)	4.62±1.99	5.02±1.04	4.90±1.93	3.73±1.34	0.25
R (min)	5.16±2.00	5.60±1.16	5.60±2.29	4.23±1.93	0.18
K (min)	1.74±0.34	1.92±0.75	2.23±0.84	1.90±0.36	0.59
Angle (degree)	65.86±4.39	64.50±8.26	60.93±9.26	63.97±4.73	0.44
MA (mm)	59.16±2.28	58.58±2.36	51.40±4.31	54.17±6.90	0.11
G (d/s)	7.20±0.71	7.10±0.73	5.40±0.91	6.00 ±1.55	0.11
LY30 (%)	0.36±0.80	0.32±0.51	0.37±0.55	0.47±0.72	0.83
CI	0.42±1.35	−0.10±1.47	0.30±0.57	0.00±1.56	0.71

This table shows the mean ± standard deviation values for each parameter in the clopidogrel and placebo groups on days 0 and 7. The *p*-value reflects the significance of differences between the two groups over time. TEG, thromboelastography; SP, split point; R, reaction time; K, clot kinetics; MA, maximum amplitude; G, global clot strength; and CI, coagulation index.

The calculated platelet mapping parameter incorporates all three maximal amplitude values and represents the percentage reduction in the MA attributed to ADP receptor inhibition. This parameter varied widely among the dogs. The mean ± SD for the clopidogrel-treated dogs was 72.06 ± 18.40% on day 0 and was 99.22 ± 0.83% on day 7. The median value for the placebo group was 69.23 ± 14.76% on day 0 and 65.80 ± 21.28% on day 7. Notably, significant differences in inhibition were observed between the groups on days 0 and 7 after drug administration.

### 3.3 Flow cytometric analyses of activation parameters on agonist-stimulated platelets

Flow cytometry revealed significant inter-group differences in the MFI of CD62P-PE positive platelets after ADP and epinephrine stimulation. The clopidogrel group demonstrated a larger decrease in MFI from 61.74 ± 37.26 on day 0 to 20.40 ± 16.26 on day 7 (*p* < 0.05) whereas the placebo group did not show any difference.

**Figure 3:**
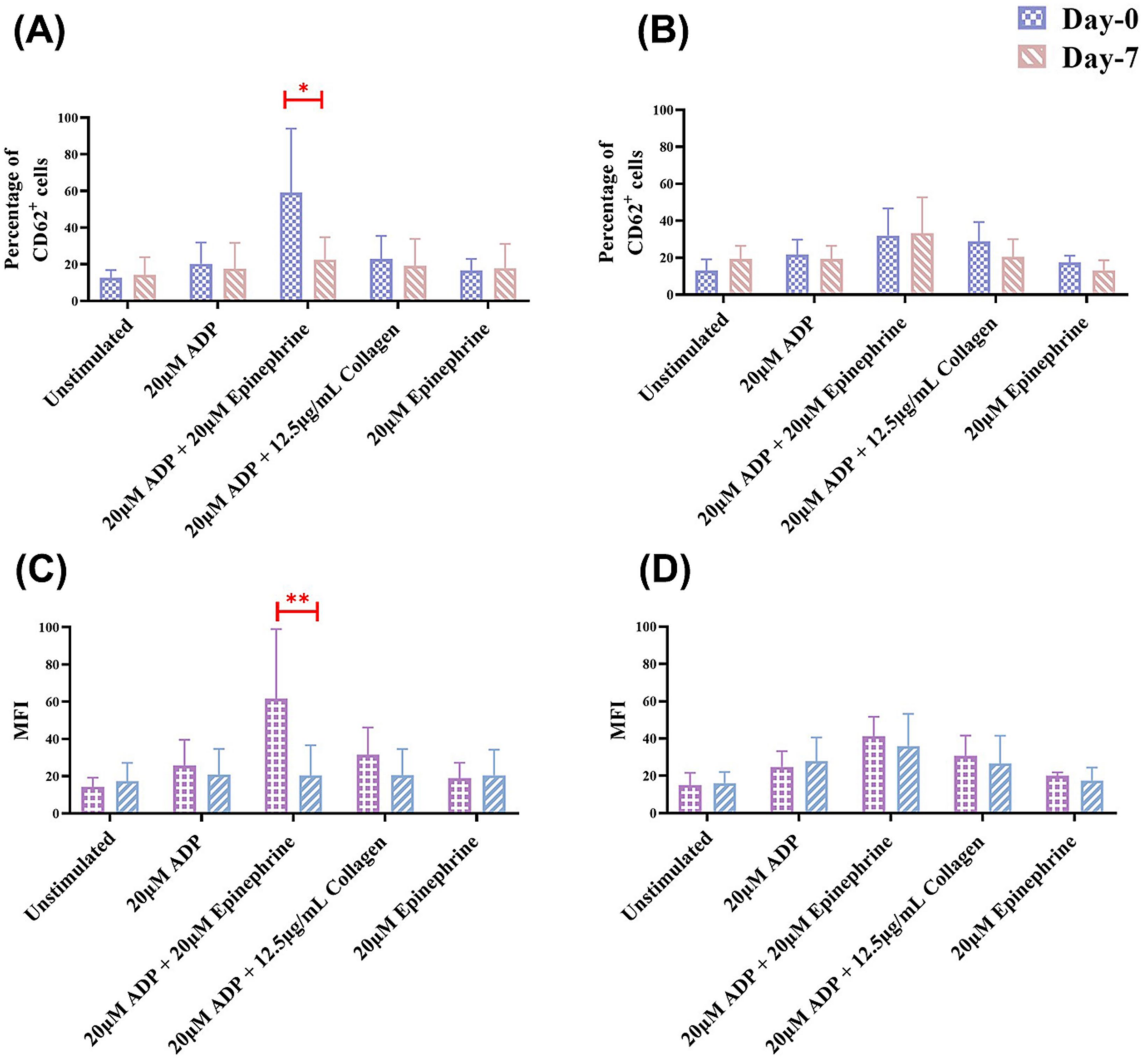
Results of the mean percentage of CD62P-positive cells and mean fluorescence intensity (MFI) in flow cytometry within groups over time. This figure shows the mean percentage of CD62P-positive cells in flow cytometry analysis for **(A)** the clopidogrel and **(B)** placebo groups on days 0 and 7. It also shows the results of the MFI in flow cytometry for **(C)** the clopidogrel group and **(D)** placebo group over time. Various conditions are shown, including unstimulated samples and samples stimulated with 20 μM ADP, 20 μM ADP + 20 μM epinephrine, 20 μM ADP + 12.5 μg/mL collagen, and 20 μM epinephrine. ^*^p < 0.05 and ^**^p < 0.01, indicating a significant difference between results for days 0 and 7 within each group. ADP, adenosine diphosphate.

Additionally, intra-group comparisons revealed significant reductions in platelet reactivity over time in the clopidogrel group, especially for the ADP and epinephrine stimulation conditions, where the *p*-value for the inter-group change was highly significant (*p* < 0.01) (Figure 3). In contrast, no statistically significant changes were observed within the placebo group over the 7 days for any condition. When the changes in platelet activation between the clopidogrel and placebo groups at two-time points (days 0 and 7) were evaluated, the clopidogrel group demonstrated a significant reduction in CD62P-positive events for ADP and epinephrine stimulated condition (57.93 ± 32.66% on day 0 to 22.63 ± 12.15% on day 7, *p* = 0.04) compared to placebo group (32.03 ± 14.71% on day 0 to 33.37 ± 19.37% on day 7).

Additionally, intergroup comparisons revealed a statistically significant reduction in the platelet activation over time in the clopidogrel group after ADP and epinephrine stimulation, with highly significant *p*-values of < 0.05 (Figure 3).

### 3.4 Clinicopathologic data

Comprehensive evaluations revealed no significant differences in the CBC variables and fibrinogen concentration between the groups at any time point.

Serum biochemical analyses indicated no clinically relevant deviations from the baseline values on day 7, regardless of whether the dogs were treated with clopidogrel. All results remained within the laboratory reference ranges (Table 2).

**Table 2:** Results of blood analysis before (Day 0) and after (Day 7) treatment in the clopidogrel and placebo groups.

Variable	Clopidogrel		Placebo		*p* value
	Day 0	Day 7	Day 0	Day 7	
HCT (%)	44.30±4.45	43.98 ±4.49	45.83±2.57	45.83±4.19	0.89
PLT (10×9/L)	252.20±43.11	258.40±26.60	256.00±38.04	297.00±45.00	0.24
Fibrinogen (mg/dl)	181.80±13.24	177.40±15.32	186.00±9.17	183.67±10.21	0.83
WBC (10×9/L)	6.82±1.19	7.11±1.48	6.43±0.71	7.33±0.67	0.88
ALT (U/L)	132.60±15.90	132.00±44.62	128.33 ±21.46	117.67±24.54	0.80
ALP (U/L)	55.40±20.02	50.40±8.68	53.67±19.04	55.33±12.66	0.43
GGT (U/L)	4.60±2.70	3.00±4.12	2.67±3.06	3.00±1.73	0.34

This table shows the mean ± standard deviation values for each parameter in the clopidogrel and placebo groups on days 0 and 7. The *p*-value reflects the significance of differences between the two groups over time. HCT, Hematocrit; PLT, Platelet count; ALT, Alanine aminotransferase; ALP, Alkaline phosphatase; GGT, Gamma-glutamyl transferase; WBC, White blood cell.

In the clopidogrel treatment group, ALT activity showed no significant changes from 132.60 ± 15.90 U/L on day 0 to 132.00 ± 44.62 U/L on day 7. The ALP levels slightly decreased from 55.40 ± 20.02 U/L to 50.40 ± 8.68 U/L, which was also not statistically significant. Additionally, the GGT levels remained stable at 4.60 ± 2.70 U/L on day 0 and 3.00 ± 4.12 U/L on day 7.

### 3.5 Individual response to clopidogrel and treatment-induced platelet function reduction

**Figure 4:**
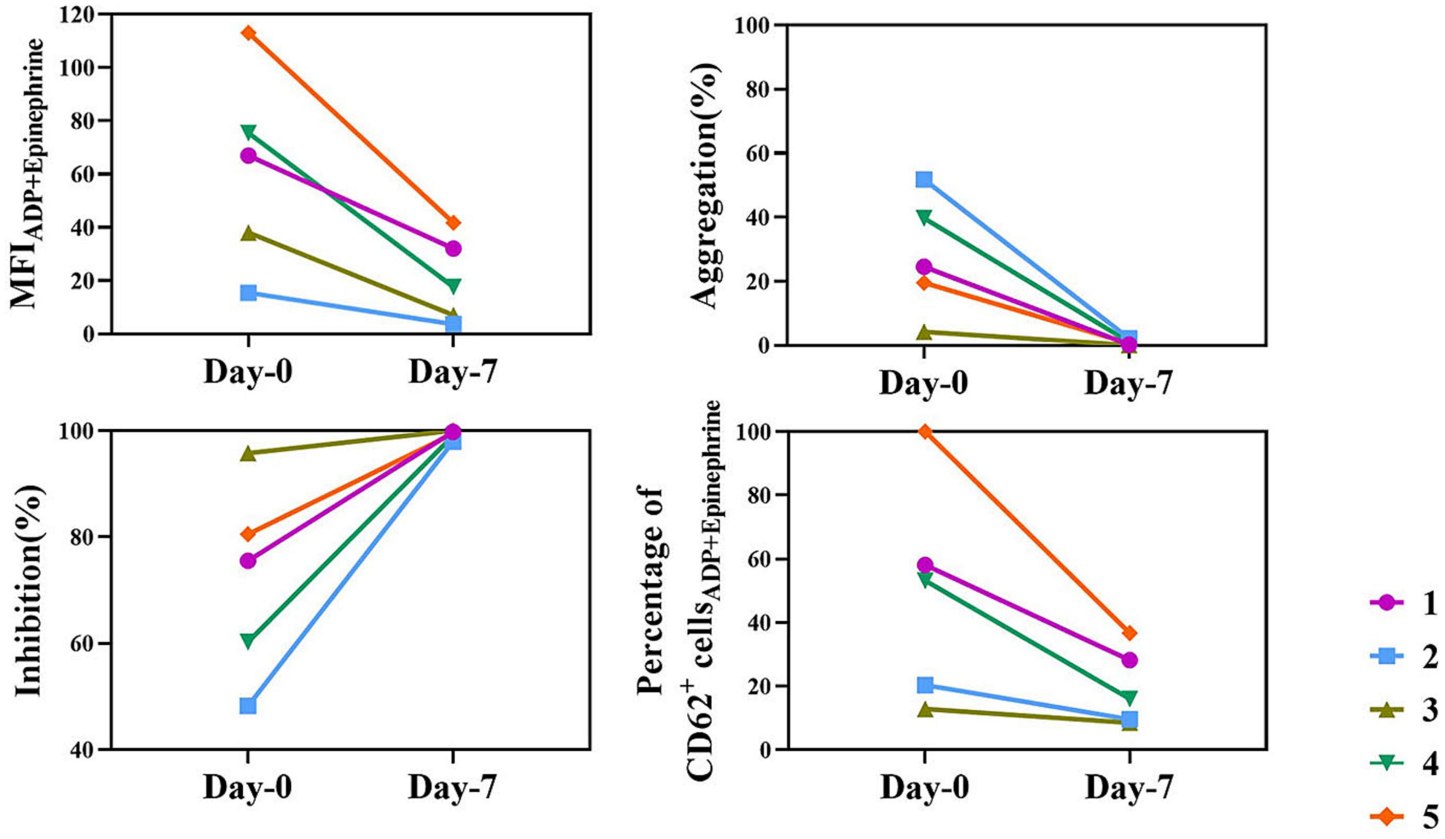
Individual results of platelet mapping TEG and flow cytometry in the clopidogrel group. This figure shows platelet mapping TEG and flow cytometry parameters for each dog in the clopidogrel group on days 0 and 7. Each line represents a different individual (identified by different colors and symbols). The parameters include MFI for ADP + epinephrine stimulation, aggregation (%), inhibition (%), and the percentage of CD62P-positive cells in flow cytometry. Despite baseline differences, all dogs exhibited a marked and consistent reduction in platelet function following clopidogrel treatment. ADP, adenosine diphosphate; MFI, mean fluorescence intensity; TEG, thromboelastography.

Figure 4 illustrates the changes in platelet function before and after clopidogrel treatment, as measured by TEG platelet mapping (MA values) and flow cytometry (CD62P expression) in response to ADP and epinephrine stimulation. Although baseline values varied among individuals, all dogs exhibited a marked and consistent reduction in platelet activation after treatment.

## 4 Discussion

Clopidogrel effectively suppressed platelet function across all subjects regardless of initial reactivity. These findings support the adequacy of low-dose clopidogrel in achieving significant antiplatelet effects, while also highlighting the importance of monitoring platelet function to tailor therapy to individual responses. Despite the interindividual variability in the baseline measurement, all dogs had significant reductions in ADP-induced platelet aggregation following drug administration in this study. Although platelet inhibition with or without a loading dose was not compared, sufficient antiplatelet effects are maintained without a loading dose in this study. When clopidogrel was administered, no other systemic effects were observed, and PLTs remained stable, indicating specific targeting of platelet activation. Additionally, there were no significant changes in the red or white blood cell counts due to the drug. Clopidogrel is a prodrug required to undergo hepatic metabolism to be converted into its active form to exert its effects. Therefore, the liver enzyme concentrations were measured to assess their potential impact on the liver, and no significant differences were found. No adverse reactions related to platelet dysfunction were observed throughout the study, suggesting that clopidogrel is safe for reducing platelet aggregation in dogs at the tested doses.

Platelet function tests measure platelet activation and are used for screening, diagnosis, and monitoring therapies. They help optimize antiplatelet treatment by assessing medication effectiveness. However, few tests can fully evaluate platelet pathways due to variability in methods. TEG assesses clot formation and platelet function in dogs, offering insights into clotting and bleeding tendencies, and aiding in treatment plans, though it has limitations like false results related to HCT and fibrinogen levels (21–23). Platelet mapping TEG, a variant for antiplatelet therapy, measures platelet function and coagulation with low variation, but further studies are needed to establish its role (24, 25).

Among the various methods available, we chose to perform a comparative analysis using TEG and flow cytometry. TEG offers a comprehensive overview of clot formation and bleeding tendencies, whereas flow cytometry allows for a more detailed investigation of cellular platelet characteristics (26). Despite the different mechanisms used to assess platelet function, platelet-mapping TEG and flow cytometry yielded broadly similar patterns of inhibition. TEG assesses clot dynamics through viscoelastic measurements, which is particularly valuable in clinical settings. In contrast, flow cytometry evaluates surface marker expression in response to specific agonists, allowing the detection of subtle changes in the platelet activation status through specific markers. The integration of TEG and flow cytometry not only enhances the ability to assess platelet function but also enables clinicians to better anticipate potential complications related to antiplatelet therapy. For instance, by combining clot dynamics data from TEG with cellular activation insights from flow cytometry, veterinarians can be better positioned to identify patients with a higher risk of bleeding or thrombosis more accurately. This dual approach is especially important in managing complex cases where a one-size-fits-all approach to therapy may fail to address the unique biological factors affecting individual patients. In this study, while the two techniques yielded broadly similar patterns of inhibition, this similarity should be interpreted with caution, given their different mechanisms of assessment. TEG effectively demonstrated overall clotting dynamics while flow cytometry characterized platelet activation in response to specific agonists such as ADP and epinephrine. This suggests complementary utility but does not necessarily imply both are required in routine practice. We acknowledge that this study did not evaluate individualized bleeding risk or provide a detailed characterization of intracellular platelet signaling pathways. Rather, the flow cytometric analysis focused on surface expression of activation markers in response to selected agonists. These findings underscore the complementary strengths of these two methods in assessing platelet function, which could be beneficial for optimizing antiplatelet therapy.

A prospective study demonstrated that oral clopidogrel effectively inhibited canine platelet aggregation at a dose of 1 mg/kg administered orally every 24 h without a loading dose (6, 27). Previous studies have suggested a loading dose ranging from 4 to 10 mg/kg on the first day of treatment (28, 29), but our findings support significant antiplatelet effects even at the tested doses. The pharmacokinetics of clopidogrel remain poorly understood, and the variability of the time required to achieve full platelet inhibition may justify the use of a loading dose when initiating therapy. Although all dogs responded to clopidogrel with a significant reduction in platelet activity, some variability in baseline values and the degree of inhibition was noted, which may inform future considerations for individualized therapy in certain clinical contexts. Several studies in human and veterinary medicine have been conducted to identify the optimal clopidogrel dosage to maintain thromboprophylactic effects within the therapeutic window. However, variability in individual responses indicate that genetic factors, metabolic pathways, and other underlying factors may significantly influence the antiplatelet effects of clopidogrel. These variations made the identification of an effective dosage for treatment complicated. Therefore, recent research, including the current study, has aimed to assess the therapeutic effects of clopidogrel on individuals through various platelet function tests. Monitoring with platelet function assessments is recommended to verify and quantify the effect of the drug, as practiced in human medicine, where such evaluations ensure that thromboprophylactic effects remain within the therapeutic window.

Our study has some limitations. First, we used healthy dogs that showed no signs of disease or hypercoagulability; however, the effect of the drug on patients with critical illness or those with other medications who truly require clopidogrel is uncertain. Dogs that are hypercoagulable or have hyperactive platelets due to naturally occurring disorders may respond differently to clopidogrel. Secondly, clopidogrel is not available in an injectable form, and its use is restricted to dogs that cannot tolerate oral administration. The pharmacodynamics and pharmacokinetics of clopidogrel can be altered by medications that inhibit hepatic CYP enzymes, such as rifampin or cimetidine (30, 31). Understanding these pharmacological interactions is crucial for guiding future applications of clopidogrel in veterinary practice. Third, the sample size calculation indicated that five dogs per group were adequate to detect significant differences during the course of treatment. Significant differences were observed between the treatment groups in this study, but a larger sample size may have yielded different results, especially in the clopidogrel group. Fourth, we assessed the platelet function at only two time points during drug administration. Evaluation at additional time points may have provided a more comprehensive understanding of drug-induced platelet dysfunction, although changes in platelet function during antiplatelet therapy tend to be gradual. Finally, as mentioned earlier, a comparison between dogs receiving a loading dose and those not receiving a loading was not investigated with respect to plasma drug concentrations and platelet inhibition. This investigation should be confirmed in a further study.

In conclusion, this study demonstrated that clopidogrel can provide adequate antiplatelet effects in dogs at the studied doses without the need for a loading dose, thus reducing the risk of adverse effects from unexpectedly high drug concentrations. However, individual responses to clopidogrel can vary significantly, owing to genetic and metabolic differences among dogs. Although evidence-based dosage guidelines for antiplatelet and anticoagulant therapies are well established in human medicine, similar guidelines are still under development in veterinary medicine. Drug monitoring is crucial for optimizing antithrombotic therapy and ensuring appropriate intensity, while minimizing the risk of bleeding complications. The antiplatelet effects of clopidogrel do not directly correlate with plasma concentrations due to variations in hepatic metabolism, highlighting the need for individualized therapeutic approaches.

Furthermore, TEG and flow cytometry provide clinically accessible methodologies that are highly applicable to clopidogrel therapy monitoring. Their potential to become the gold standard for platelet function assessment is substantiated by their ability to provide comprehensive insights into platelet function. Future research should focus on these modalities as they hold significant promise for advancing antiplatelet management in veterinary medicine. Such an exploration will be critical for refining therapeutic strategies and improving patient outcomes. This will bridge the gap between current clinical practice and optimal care in veterinary and human medicine.

## Data Availability

The original contributions presented in the study are included in the article/Supplementary material, further inquiries can be directed to the corresponding authors.

## Supplementary material

SUPPLEMENTARY FIGURE 1 Flow cytometric analysis of platelet activation. **(A)** Platelet populations were gated based on forward scatter (FSC-H) and side scatter (SSC-H) to exclude debris. **(B)** CD61-APC-positive events were selected to specifically identify platelets. **(C)** Expression of CD62P, a marker of platelet activation. The x-axis indicates CD62P-FITC fluorescence intensity (log scale), and the y-axis indicates event counts. The gray histogram represents the isotype control, the dashed line represents unstimulated platelets, and the solid line represents platelets stimulated with ADP (left) or ADP + epinephrine (EPI) (right). The horizontal bar indicates the CD62P-positive population.
