# Supplementary figures and images for: Evaluation of clopidogrel impact on canine platelet function using flow cytometry and thromboelastography platelet mapping
Source: Front Vet Sci. 2025 Jun 5;12:1555641. doi: 10.3389/fvets.2025.1555641 (PMC12176556; doi:10.3389/fvets.2025.1555641)

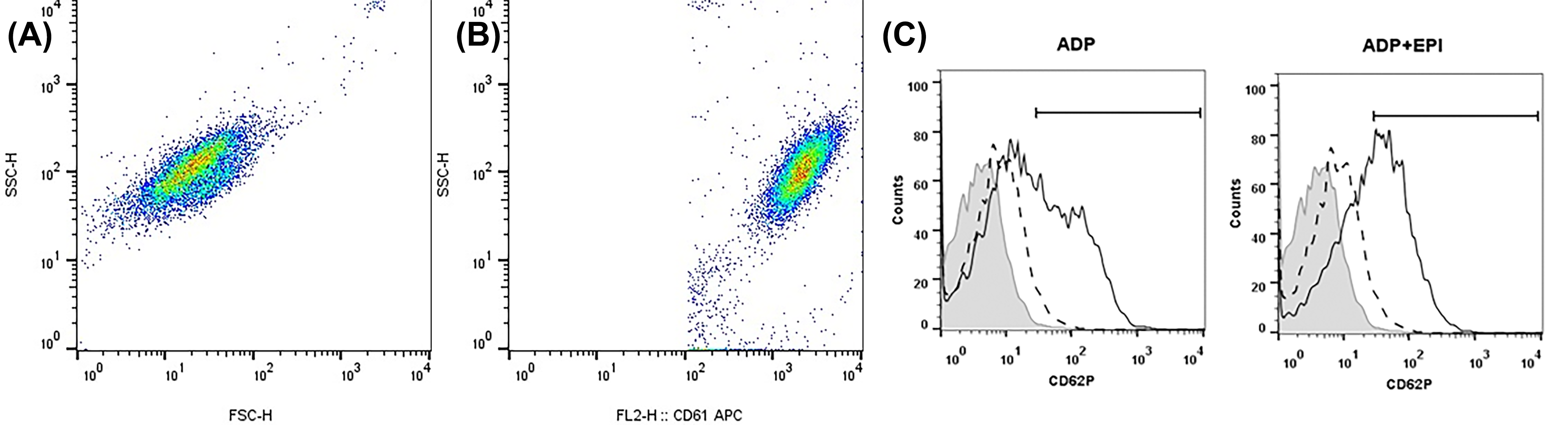

Supplement: SUPPLEMENTARY FIGURE 1 — Flow cytometric analysis of platelet activation. (A) Platelet populations were gated based on forward scatter (FSC-H) and side scatter (SSC-H) to exclude debris. (B) CD61-APC-positive events were selected to specifically identify platelets. (C) Expression of CD62P, a marker of platelet activation. The x-axis indicates CD62P-FITC fluorescence intensity (log scale), and the y-axis indicates event counts. The gray histogram represents the isotype control, the dashed line represents unstimulated platelets, and the solid line represents platelets stimulated with ADP (left) or ADP + epinephrine (EPI) (right). The horizontal bar indicates the CD62P-positive population. [file Image_1.TIF]
